# Prognostic value of prehospital single measurement of N-terminal pro-brain natriuretic peptide and troponin T after acute ischemic stroke

**DOI:** 10.1186/cc9737

**Published:** 2011-03-11

**Authors:** S Grmec, B Kit, E Hajdinjak

**Affiliations:** 1 ZD dr. Adolfa Drolca Maribor, Slovenia

## Introduction

The association between levels of N-terminal fragment of pro-brain natriuretic peptide (NT-proBNP), troponin T and prognostic outcomes in patients after ischemic stroke were tested. Acute-phase levels of NT-pro-BNP and troponin T have been associated with mortality when measured in patients with an acute ischemic stroke. However, the value of pre-interventional levels of NT-pro-BNP and troponin T measured in the field as a prognosticator of in-hospital mortality after ischemic stroke is limited.

## Methods

This prospective study was performed in the Center for Emergency Medicine Maribor, Slovenia from June 2006 to May 2010. Blood samples for NT-proBNP and troponin T levels were collected in the prehospital setting and examined with a portable Cardiac Raeder device after acute ischemic stroke in 106 consecutive patients (204 patients with acute stroke were excluded). ECG and other variables previously associated with severity of stroke were also recorded and assessed as independent predictors of inpatient mortality.

## Results

Troponin T was elevated (>0.04 μg/l) in 16 out of 106 patients (15.1%). Twenty-three patients died in the hospital. Raised troponin T occurred in eight patients in this group (8/23; 34.8%) versus eight patients (8/83; 9.6%) who survived until hospital discharge (*P *< 0.01). NT-pro-BNP concentrations were significantly higher in decedents (508 pg/ml, 10th to 90th percentiles 98 to 3,000) than in the 83 survivors (153 pg/ml, 10th to 90th percentiles 49 to 690, *P *< 0.001). In logistic regression analyses, a rise in troponin T (odds ratio, 1.8; 95% CI, 1,03 to 8.43, *P *< 0.01) and NT-pro BNP (odds ratio, 5.80; 95% confidence interval, 1.33 to 22.72, *P *< 0.01) were significantly associated with a poor short-term outcome.

## Conclusions

The NT-pro-BNP and troponin T concentrations measured during the prehospital phase of care after acute ischemic stroke are strong predictors of in-hospital mortality.
